# The 'Switch’ study protocol: a randomised-controlled trial of switching to an alternative tumour-necrosis factor (TNF)-inhibitor drug or abatacept or rituximab in patients with rheumatoid arthritis who have failed an initial TNF-inhibitor drug

**DOI:** 10.1186/1471-2474-15-452

**Published:** 2014-12-23

**Authors:** Nuria C Navarro Coy, Sarah Brown, Ailsa Bosworth, Claire T Davies, Paul Emery, Colin C Everett, Catherine Fernandez, Janine C Gray, Suzanne Hartley, Claire Hulme, Anne-Maree Keenan, Christopher McCabe, Anthony Redmond, Catherine Reynolds, David Scott, Linda D Sharples, Sue Pavitt, Maya H Buch

**Affiliations:** Leeds Institute of Rheumatic and Musculoskeletal Medicine, University of Leeds, 2nd Floor, Chapel Allerton Hospital, Leeds, LS7 4SA UK; NIHR Leeds Musculoskeletal Biomedical Research Unit, Chapel Allerton Hospital, Leeds Teaching Hospitals Trust, Leeds, LS7 4SA UK; Clinical Trials Research Unit, Leeds Institute of Clinical Trials Research, University of Leeds, Leeds, LS2 9JT UK; National Rheumatoid Arthritis Society (NRAS), Maidenhead, Berkshire SL6 3RT UK; Academic Unit of Health Economics, Leeds Institute of Health Sciences, University of Leeds, Leeds, LS2 9LJ UK; University of Alberta, 112 Street NW, Edmonton, Alberta Canada; Centre for Health Sciences Research, Leeds Institute of Health Sciences, University of Leeds, Leeds, LS2 9LJ UK; School of Medicine, University of East Anglia, Norfolk, NR4 7QN UK

**Keywords:** Rheumatoid arthritis, TNF-inhibitor, Rituximab, Abatacept, Non-responder, Biologics, Randomised clinical trial, Cost-effectiveness

## Abstract

**Background:**

Rheumatoid Arthritis (RA) is one of the most common autoimmune diseases, affecting approximately 1% of the UK adult population. Patients suffer considerable pain, stiffness and swelling and can sustain various degrees of joint destruction, deformity, and significant functional decline. In addition, the economic burden due to hospitalisation and loss of employment is considerable, with over 50% of patients being work-disabled within 10 years of diagnosis. Despite several biologic disease modifying anti-rheumatic drugs (bDMARD) now available, there is a lack of data to guide biologic sequencing. In the UK, second-line biologic treatment is restricted to a single option, rituximab. The aim of the SWITCH trial is to establish whether an alternative-mechanism-TNF-inhibitor (TNFi) or abatacept are as effective as rituximab in patients with RA who have failed an initial TNFi drug.

**Methods/Design:**

SWITCH is a pragmatic, phase IV, multi-centre, parallel-group design, open-label, randomised, controlled trial (RCT) comparing alternative-mechanism-TNFi and abatacept with rituximab in patients with RA who have failed an initial TNFi drug. Participants are randomised in a 1:1:1 ratio to receive alternative mechanism TNFi, (monoclonal antibodies: infliximab, adalimumab, certolizumab or golimumab or the receptor fusion protein, etanercept), abatacept or rituximab during the interventional phase (from randomisation up to week 48). Participants are subsequently followed up to a maximum of 96 weeks, which constitutes the observational phase. The primary objective is to establish whether an alternative-mechanism-TNFi or abatacept are non-inferior to rituximab in terms of disease response at 24 weeks post randomisation. The secondary objectives include the comparison of alternative-mechanism-TNFi and abatacept to rituximab in terms of disease response, quality of life, toxicity, safety and structural and bone density outcomes over a 12-month period (48 weeks) and to evaluate the cost-effectiveness of switching patients to alternative active therapies compared to current practice.

**Discussion:**

SWITCH is a well-designed trial in this therapeutic area that aims to develop a rational treatment algorithm to potentially inform personalised treatment regimens (as opposed to switching all patients to only one available (and possibly unsuccessful) therapy), which may lead to long-term improved patient outcomes and gains in population health.

**Trial registration:**

UKCRN Portfolio ID: 12343;ISRCTN89222125;NCT01295151

**Electronic supplementary material:**

The online version of this article (doi:10.1186/1471-2474-15-452) contains supplementary material, which is available to authorized users.

## Background

Rheumatoid Arthritis (RA) is one of the most common autoimmune diseases; a chronic, systemic, inflammatory arthritis, affecting approximately 1% of the UK adult population[1] and is the largest cause of treatable disability in the Western world[2, 3]. Patients suffer considerable pain, stiffness and swelling and if not adequately controlled, sustain various degrees of joint destruction, deformity, and significant functional decline[3]. In addition to the impact of RA on the individual, the health economic and societal burden is considerable, due to hospitalisation and loss of employment with over 50% of patients work-disabled within 10 years of diagnosis[4].

RA is also associated with a significant increase in mortality, up to three-fold compared to the general population[5] with the standardised mortality rates (SMR) in severe cases, described as comparable to Non-Hodgkin’s lymphoma, triple vessel coronary artery disease and cerebrovascular disease[6]. The increased mortality is largely due to increased frequency of premature cardiovascular disease (CVD)[7], which constitutes up to 40% of mortality in RA patients and is as high as that of patients with other major CVD risk factors such as Type 2 diabetes mellitus[8]. This appreciation has further highlighted the importance of ensuring optimal and effective disease control.

Expedient implementation of disease modifying anti-rheumatic drug (DMARD) therapy is the cornerstone of management of RA. Nevertheless, it has become clear that poor response (even if initially effective) remains a feature with most DMARDs over time. In addition, a high incidence of toxicity has been observed with these drugs[9]. Such obstacles to therapy combined with data suggesting limited alteration in long-term outcome even in those showing response has argued for more effective therapeutic options[10].

This unmet clinical need fuelled research into RA, which led to significant advances in our understanding of RA by the 1990s, with an appreciation of the role of excess pro-inflammatory cytokines, in particular tumour necrosis factor (TNF) in driving RA pathogenesis[11]. Following *in vitro* and *in vivo* work, the most compelling evidence for a key role for TNF-inhibitor (TNFi) stemmed from studies where marked clinical benefit was observed in patients with RA treated with chimeric TNF-alpha monoclonal antibodies[12]. The subsequent introduction of several costly but highly effective TNFi therapies marked the start of a new era in biologic DMARD (bDMARD) drug development for RA[13–15].

### TNF-inhibitors

Cochrane reviews provide clear evidence that the licensed TNFi drugs (etanercept, infliximab, adalimumab, certolizumab and golimumab) produce better outcomes in RA compared with placebo or treatment with conventional DMARDs[16–19]. All these are in the same class of drug i.e. TNFi, but differ in several respects:

i. Molecule type [infliximab, chimeric (mouse-human) monoclonal antibody; adalimumab, humanised and golimumab, fully human monoclonal antibody; certolizumab, PEGylated Fab fragment of a humanised monoclonal antibody to TNF and etanercept, fusion protein];
ii. Target (etanercept binds both TNF-alpha and another cytokine, lymphotoxin-alpha);
iii. Binding affinity to TNF [20];
iv. Mechanism of drug action [20–22];
v. Route of administration (all subcutaneous except for infliximab);
vi. Frequency of administration.

Despite the extensive benefits of TNF-directed biologic therapies, a significant proportion of RA patients fail to achieve sufficient response[23]. Two broad approaches can be employed to manage initial TNFi non-response; switching to an alternative TNFi therapy or use of another mechanism agent. Of the latter, rituximab, a B-cell depleting therapy, abatacept, and more recently, tocilizumab, have been licensed, although only rituximab is currently approved by the National Institute for Health and Care Excellence (NICE) at the TNFi-failure stage[24].

### Switching between TNF-inhibitors

Current NICE guidance does not permit switching to an alternative TNFi as a second-line biologic therapy choice unless rituximab +/- methotrexate is contraindicated. Several early phase, uncontrolled studies and an initial, small, randomised study suggested benefit in switching between TNFi agents[25–35]. A report of high ACR20 responses on an alternative TNFi agent in specific sub-group of patients[27] also indicates the potential value of and the need to explore this approach further. The rationale and argument for switching between different TNFi drugs was strengthened by a large, randomised industry-led efficacy study comparing golimumab with placebo. This phase III study of 461 patients who had previously received and either failed or were intolerant to one or more TNFi were randomised to placebo, subcutaneous golimumab 50 mg or 100 mg 4-weekly. Significantly higher ACR20 response rates at week 14 were observed in the 50 mg and 100 mg golimumab groups compared to placebo group (35% and 38% versus 18% respectively)[36].

A key benefit of the TNFi is their suitability in both seropositive and seronegative disease [to rheumatoid factor (RF) +/- anti-citrullinated peptide antibody (ACPA)]. This is in contrast with data implying the influence of antibody status and response rates in patients treated with rituximab (particularly at the TNFi-failure stage, see below) due to its distinct target and rationale for use (rituximab depletes the autoantibody producing B-cells)[37, 38]. It is therefore important not to prematurely discount an alternative TNFi drug as an effective therapeutic option, particularly in the context of such resistant and aggressive disease cohorts. In addition, patients with RA may have a co-existing immune-mediated inflammatory disease (for example, inflammatory bowel disease, psoriasis) that would also be amenable to treatment with a TNFi (with rituximab not as suitable and potentially toxic)[36, 39–42]. Having the option of using a second TNFi in this scenario would be clinically more appropriate than having to potentially consider two different classes of bDMARDs.

### Alternative bDMARD therapies

Industry-led efficacy studies have demonstrated benefits of rituximab, abatacept and tocilizumab after TNFi failure[43–47] although only rituximab is NICE-approved (and neither abatacept nor a TNFi has been compared to rituximab). Rituximab, however, is not appropriate for certain patients and may even lead to unpredictable responses or toxicity[48], or failure to respond (up to a third of patients). Furthermore, meta-analyses of rituximab suggests seronegative antibody status, seen in up to 25-30% of patients, appears to be associated with poorer response, particularly in the TNFi-failure trial; although this has not been formally tested[37, 44, 45, 49, 50]. Recent data on abatacept from an observational registry also suggests seropositive status may confer greater benefits to abatacept therapy[51].

A Swiss observational study[52] comprised 116 patients who had failed at least one TNFi agent and were switched to either an alternative TNFi therapy or to one cycle of rituximab with the results suggesting that rituximab was the more favourable treatment option. Aside from the small sample size, this retrospective study had several other design limitations with outcome taken from differing time-points and inclusion of all types of initial TNFi failure; in addition it was neither controlled nor randomised to treatment type. The observational studies MIRAR and SWITCH-RA have also reported the use of rituximab as a better strategy compared to an alternative TNFi drug following insufficient response to a first TNFi[53, 54]. The collaborative CERRERA registry[55, 56] has also suggested utility of rituximab but in contrast to the Swiss study, following TNFi toxicity as opposed to lack of efficacy[57]. Other observational studies comparing alternative TNFi with other bDMARDs, such as abatacept and tocilizumab as well as rituximab, also favour these therapies over the use of alternative mechanism TNFi as second line treatment[58–60]. These results have also been consolidated by recent RCTs (preliminary data)[61, 62] and meta-analyses, which have failed to demonstrate superiority of one therapy over another[63], with European recommendations also confirming all currently licensed therapies as appropriate options[64].

It therefore remains unclear how best to utilise the alternative bDMARDs described above following initial TNFi failure. It is apparent that no universally effective treatment exists with the present approach, and clinicians treating patients in the absence of sufficiently strong data is unsatisfactory. The current reality of second-line bDMARD restricted to a single option (rituximab) in the UK seriously impedes effective management. This is particularly pertinent to patient sub-groups where alternative licensed therapies may seem more appropriate (e.g. in seronegative RA, concomitant immune mediated inflammatory diseases). This poses a significant problem to the NHS and is in conflict with the patient agenda. Despite several treatment options now being available, no good quality head-to-head comparisons investigating the efficacy of sequential biologic treatments have been conducted to date.

The SWITCH trial aims to provide clear guidance to clinicians. The results of this study will enable the development of a rational treatment algorithm and should enable more judicious and cost-effective management; in particular it will potentially allow personalised treatment regimens as opposed to switching all patients to only one available (and potentially unsuccessful) therapy, potentially leading to long-term net-benefits and improved patient outcomes.

Whilst more recent technology appraisal permits the use of abatacept, a T-cell co-stimulation blockade agent, and tocilizumab, an interleukin-6 receptor monoclonal antibody, as first-line biologic together with TNFi[65], TNFi, remains the predominant first-line bDMARD currently prescribed in the UK[66].

## Methods/Design

### Trial aims and objectives

The general aim of the trial is to compare alternative-mechanism TNFi to rituximab, and abatacept to rituximab in terms of disease response, quality of life, cost-effectiveness, toxicity and safety over a 12-month (48 weeks) period. Each of the two comparisons (TNFi vs. rituximab and abatacept vs. rituximab) is considered to be of interest independently, and the trial aims to establish non-inferiority; therefore no adjustments for multiple comparisons have been planned.

### Primary objective

To establish whether an alternative-mechanism-TNFi or abatacept are non-inferior to rituximab in terms of disease response at six months (24 weeks) post randomisation.

### Secondary objectives

- To compare alternative-mechanism-TNFi and abatacept to rituximab in terms of disease response, quality of life, toxicity, safety, structural and bone density outcomes (in terms of plain radiography and bone densitometry score) over a 12-month (48 weeks) period.
- To undertake an evaluation of the cost-effectiveness of switching patients to an alternative-mechanism TNFi, abatacept or rituximab.

### Exploratory objectives

- To determine the optimal sequence of treatments by assessing whether response to the second treatment in patients with RA is related to the initial failed TNFi (TNFi monoclonal or TNF receptor fusion protein).
- To evaluate whether the response to the second treatment (alternative mechanism TNFi, abatacept or rituximab) is related to whether the patient was a primary (no initial response) or secondary (loss of an initial) response failure to their initial TNFi.
- To ascertain whether seropositive and seronegative (to rheumatoid factor +/- anti-citrullinated peptide antibody) RA patients behave differently in their response and disease outcome measures in the three treatment arms, particularly in the comparisons with rituximab.

### Trial design

**Figure 1:**
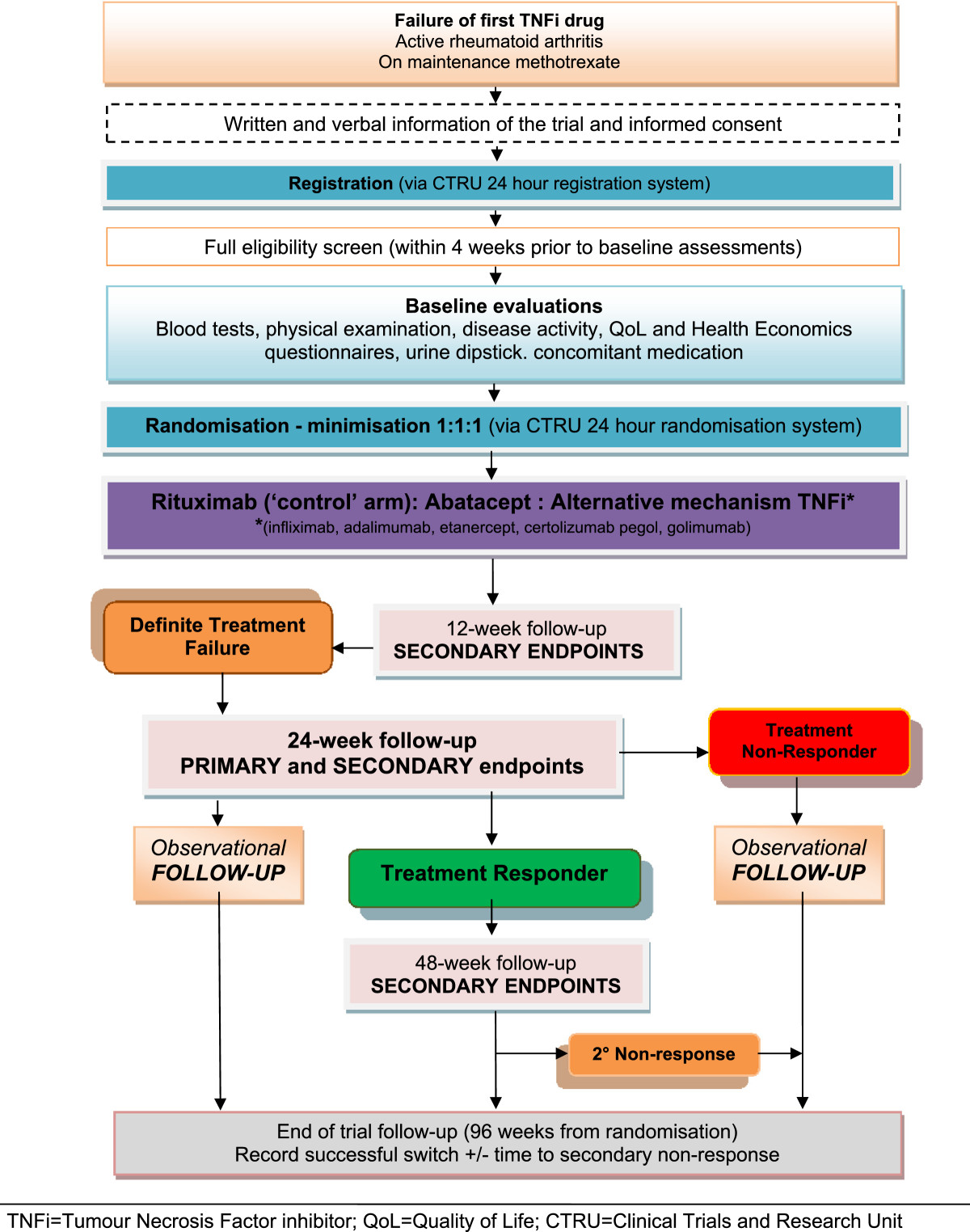
**Switch trial flow diagram.**

SWITCH is a pragmatic, phase IV, multi-centre, parallel-group, open-label, RCT comparing alternative mechanism TNFi with rituximab, and abatacept with rituximab in a total of 477 patients with rheumatoid arthritis who have failed an initial TNFi drug. Participants will be randomised to receive one of the following for a maximum of 48 weeks (interventional phase):

1. Alternative mechanism TNFi:OR
  - Etanercept if initial failure to a monoclonal antibody (infliximab, adalimumab, certolizumab or golimumab)
  - Infliximab, adalimumab, certolizumab or golimumab if initial failure to the receptor fusion protein etanercept (choice of TNFi at investigator’s discretion)
2. Abatacept
3. RituximabAll participants will subsequently be followed up from week 48 for a maximum of 96 weeks to the end of the trial, which constitutes the observational phase (see Figure  1).

### Eligibility

The British Society of Rheumatology (BSR) provides guidelines on the use of TNFi[67]. These guidelines include important exclusion criteria that are adhered to in clinical practice. These will also be applied in this study. The inclusion and exclusion criteria for randomisation into this study are detailed in Table 1.

**Table 1:** **Eligibility criteria for randomisation into the Switch trial**

	INCLUSION CRITERIA
1	Male and female subjects aged ≥18 years at the time of signing the Informed Consent Form.
2	Patients with a diagnosis of rheumatoid arthritis as per the ACR/EULAR 2010 classification criteria confirmed at least 24 weeks prior to the screening visit.
3	Patients who have failed conventional DMARD therapy as per NICE/BSR Guidelines i.e. failure of at least 2 DMARDS including methotrexate.
4	Patients with persistent RA disease activity despite having been treated with a current initial TNFi agent for at least 12 weeks. Active RA defined as*: a. Primary non-response: failing to improve DAS28 by > 1.2 or failing to achieve DAS28 ≤ 3.2 within the first 12 to 24 weeks of starting the initial TNFi.
	● This may include patients that have shown a reduction in DAS28 of >1.2 but still demonstrate unacceptably high disease activity in the physician’s judgement with evidence of an overall DAS28 of ≥3.2.
	**OR**
	b. Secondary non-response: defined as inefficacy to first TNFi (having demonstrated prior satisfactory response) as per clinician judgement; with intolerance *not* the reason for cessation of first TNFi.
5	Methotrexate dose stable for 4 weeks prior to the screening visit and to be continued for the duration of the study.
6	Patients on NSAIDs and/or corticosteroids (oral prednisolone not exceeding 10 mg daily) who have been on an unchanged regimen for at least 4 weeks prior to the screening visit and are expected to remain on a stable dose until the baseline assessments have been completed.
7	Provided written informed consent prior to any trial-specific procedures.
	*These criteria are consistent with BSR guidelines
**EXCLUSION CRITERIA**	
	*General*
1	Major surgery (including joint surgery) within 8 weeks prior to screening or planned major surgery within 52 weeks following randomisation.
	*Study Specific*
2	Patients with inflammatory joint disease of different origin, mixed connective tissue disease, Reiter’s syndrome, psoriatic arthritis, systemic lupus erythematosus, or any arthritis with onset prior to 16 years of age.
3	Patients receiving doses of prednisolone > 10 mg/day within the 4 weeks prior to the screening visit.
4	Patients receiving intra-articular or intra-muscular steroid injections within 4 weeks prior to the screening visit.
	*Excluded Previous or Concomitant Therapy:*
5	Patients who have previously received more than 1 TNFi drug OR any other biological therapy for the treatment of RA.
6	Patients unable or unwilling to stop treatment with a prohibited DMARD (i.e. synthetic DMARD aside from MTX e.g. oral or injectable gold, chloroquine, hydroxychloroquine, cyclosporine, azathioprine, leflunomide, sulphasalazine) prior to the start of protocol treatment.
7	Treatment with any investigational drug in the last 12 weeks prior to the start of protocol treatment.
	*Exclusions for general safety - These criteria should be considered in the context of BSR guidance*[44]*.*
8	Patients with other co-morbidity including acute, severe infections, uncontrolled diabetes, uncontrolled hypertension, unstable ischaemic heart disease, moderate/severe heart failure (Class III/IV of the New York Heart Association (NYHA) functional classification system), active bowel disease, active peptic ulcer disease, recent stroke (within 12 weeks before the screening visit), or any other condition which, in the opinion of the investigator, would put the patient at risk to participate in the study or would make implementation of the protocol difficult.
9	Patients with any major episode of infection requiring hospitalization or treatment with IV antibiotics within 12 weeks of start of treatment protocol or oral antibiotics within 4 weeks of start of protocol treatment.
10	Patients at significant risk of infection, which in the opinion of the investigator would put the patient at risk to participate in the study (e.g. leg ulceration, indwelling urinary catheter, septic joint within 52 weeks (or ever if prosthetic joint still in situ)).
11	Patients with known active current or history of recurrent bacterial, viral, fungal, mycobacterial or other infections including herpes zoster (for tuberculosis and Hepatitis B and C see below), but excluding fungal infections of nail beds as per clinical judgement.
12	Patients with untreated active current or latent tuberculosis (TB). Patients should have been screened for latent TB (as per BSR guidelines) within 24 weeks prior to the screening visit and, if positive, treated following local practice guidelines prior to the start of protocol treatment.
13	Patients with active current hepatitis B and/or C infection. Patients should have been screened for hepatitis B and C within 24 weeks prior to the screening visit and if positive, excluded from the study.
14	Primary or secondary immunodeficiency (history of or currently active) unless related to primary disease under investigation.
15	Pregnancy, lactation or women of child-bearing potential (WCBP) unwilling to use an effective birth control measure whilst receiving treatment and after the last dose of protocol treatment as indicated in the relevant Summary of Product Characteristics (SmPC)/Investigator Brochure (IB).
16	Men whose partners are of child-bearing potential but who are unwilling to use an effective birth control measure whilst receiving treatment and after the last dose of protocol treatment as indicated in the relevant SmPC/IB.
	*Laboratory value exclusions*
17	Patients with known significantly impaired bone marrow function as for example significant anaemia, leukopaenia, neutropaenia or thrombocytopaenia as shown by the following laboratory values at the time of the screening visit:
	● Haemoglobin < 8.5 g/dl
	● Platelet count < 100 x 10^9^/L
	● White blood cell count < 2.0 x 10^9^/L
	● Neutrophil count < 1 x 10^9^/L
18	Patients with known severe hypoproteinaemia at the time of the screening visit, e.g. in nephrotic syndrome or impaired renal function, as shown by:
	● Serum creatinine > 150 umol/L

### Recruitment

Participants will be recruited from multiple research sites within the United Kingdom; some of the collaborating research centres have been initially selected under the guidance of the Arthritis Research UK’s Adult Inflammatory Arthritis Clinical Study Group (AIA CSG). In addition, potentially eligible patients may also be identified via Participant Identification Centres (PICs). The identified clinicians at these PICs will refer potential participants to the research team based in one of the participating research sites for assessment and possible recruitment to the trial.

Patients will be approached during standard clinic visits for management of their disease. Alternatively, patients identified by other means (such as waiting lists, registries, review of case records) may be sent the personalised Switch Invitation letter inviting them to take part. Patients will be provided with verbal and written details about the trial (Participant Information Sheet and Informed Consent Document). Patients will have as long as they need to consider participation. Assenting patients will be invited to provide informed, written consent before being registered into the trial and formally assessed for eligibility.

### Consent to the switch trial BioBank

Patients who are eligible to take part in the trial will also be eligible to have a number of biological samples (blood and urine) taken for the Switch Trial BioBank. Participation will be discussed with patients at the same time as discussing their participation in the main trial. Patients who agree to have biological samples taken for the Switch Trial BioBank will be asked to sign an additional consent form.

### Screening and registration

Following written informed consent and prior to any trial related invasive or non-invasive procedures, patients will be registered into the study. All patients will undergo a screening assessment within 4 weeks prior to the baseline assessments to determine eligibility for the study.

### Randomisation

Following registration, confirmation of eligibility and completion of baseline assessments and questionnaires, participants will be randomised in a 1:1:1 ratio to receive alternative mechanism TNFi, abatacept or rituximab. Treatment group allocation will use minimisation incorporating a random element, via a computer-generated programme, to ensure treatment groups are well-balanced for: centre; disease duration (<5 years or ≥ 5 years); non-response (primary or secondary); rheumatoid factor status (RF seropositive or ACPA positive) or (RF seronegative and ACPA negative)). Both registration and randomisation will be performed centrally using an automated 24-hour telephone system based at the Leeds Clinical Trials Research Unit (CTRU).

Participating research sites will be required to complete a log of all patients over the age of 18 with RA who have failed an initial TNFi agent and have been considered for the trial, but not registered for screening or randomised, either because they are ineligible or because they decline participation.

### Trial Intervention

Treatment will be administered in the three arms as detailed in Table 2. Participants will receive the randomised treatment for a minimum of 24 weeks, after which, 24-week responders will continue treatment to 48 weeks. After week 48, the randomised treatment may be continued if response is maintained and local practice/guidelines permits on-going use of a non-NICE approved treatment if relevant. The observational phase constitutes the follow up period from week 49 to the end of the trial, with a maximum follow-up duration to week 96. The duration of the observational phase will therefore vary amongst the participants. After the observational phase participants will return to NHS routine care.

**Table 2:** **The three treatment arms of the Switch trial**

TREATMENT ARM		TREATMENT DESCRIPTION
Rituximab		Single dose of 1 g as an intravenous infusion to be administered at days 0 (week 0) and 15 (week 2; +5 days).
		In line with standard practice, a participant who loses an initial 6 month (week 24) response as per NICE guidance may receive a further cycle of rituximab after a minimum of 6 months following the first dose. The second cycle of rituximab will be given at a dose of 1 g x 2 intravenous infusions will be administered at a 2-week interval (+5 days).
Abatacept		Abatacept solution for subcutaneous injection: 125 mg/syringe (125 mg/mL). Abatacept will be given at a dose of 125 mg by subcutaneous injection at week 0 and once weekly thereafter for a minimum of 24 weeks.
		Supplied by Bristol-Myers Squibb free of charge. Trial supplies to be ordered by individual sites which will be responsible for ring-fencing abatacept upon receipt.
Alternative mechanism anti-TNF	Etanercept	Single dose of 50 mg etanercept by subcutaneous injection weekly for a minimum of 24 weeks (unless not tolerated).
	Adalimumab	Single dose of 40 mg adalimumab by subcutaneous injection every 2 weeks for a minimum of 24 weeks (unless not tolerated).
	Infliximab	Infliximab will be given at a dose of 3 mg/kg per intravenous infusion, administered on a day-case unit or equivalent. The intravenous infusions will be administered at week 0, 2 (+/- 2 days), 6 (+/- 2 days) and then 8-weekly thereafter (+/- 7 days) for a minimum of 24 weeks.
	Certolizumab Pegol	Single dose of 400 mg by subcutaneous injection at weeks 0, 2, 4 and then at a dose of 200 mg every 2 weeks thereafter for a minimum of 24 weeks.
		Certolizumab pegol will be available free of charge for the first 12 weeks of protocol treatment if supplied by UCB Pharma through their RA Patient Access Scheme.

Golimumab Single dose of 50 mg by subcutaneous injection every 2 weeks for a minimum of 24 weeks.

Participants who, in the investigator’s opinion, demonstrate an unacceptably high level of disease activity prior to week 24 may discontinue treatment if clinically indicated. These participants will be followed up as part of the observational phase of the trial. The DAS28 score[68] obtained at week 24 will be used for the primary endpoint.

### Assessments, samples and data collection

**Figure 2:**
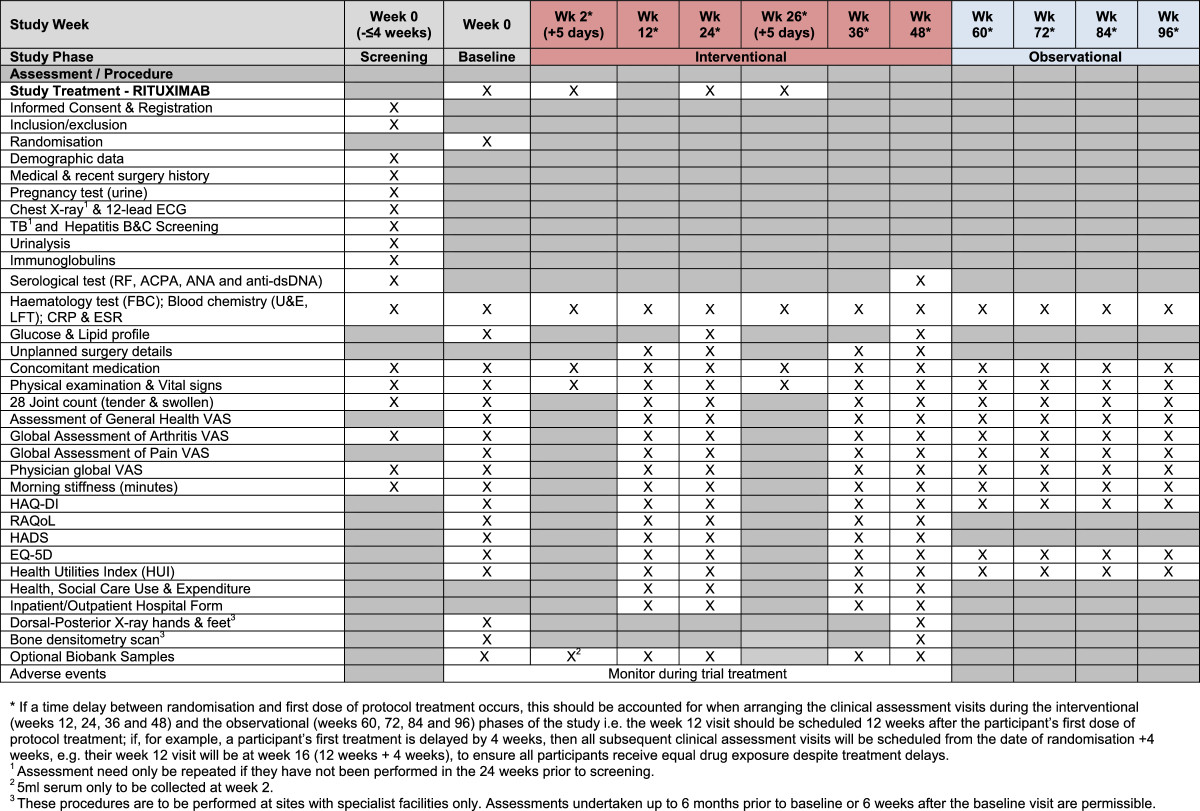
**Schedule of events for rituximab.**

**Figure 3:**
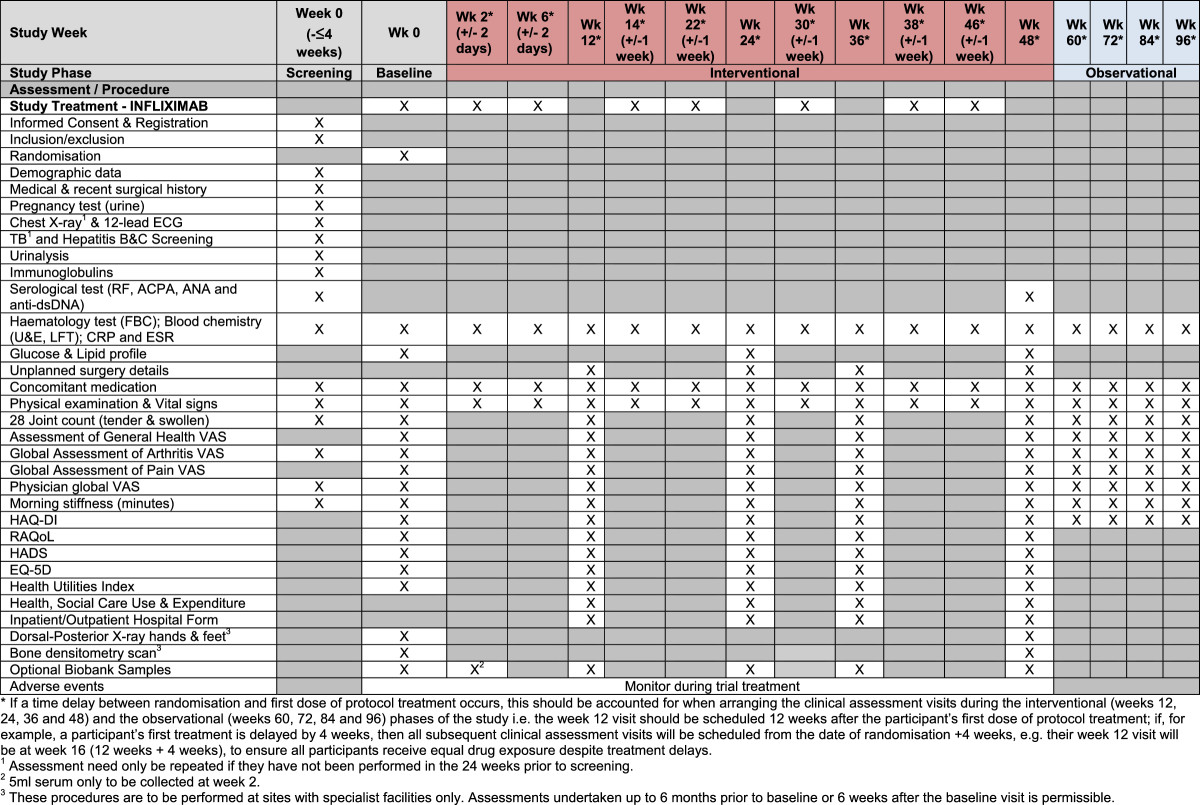
**Schedule of events for infliximab.**

**Figure 4:**
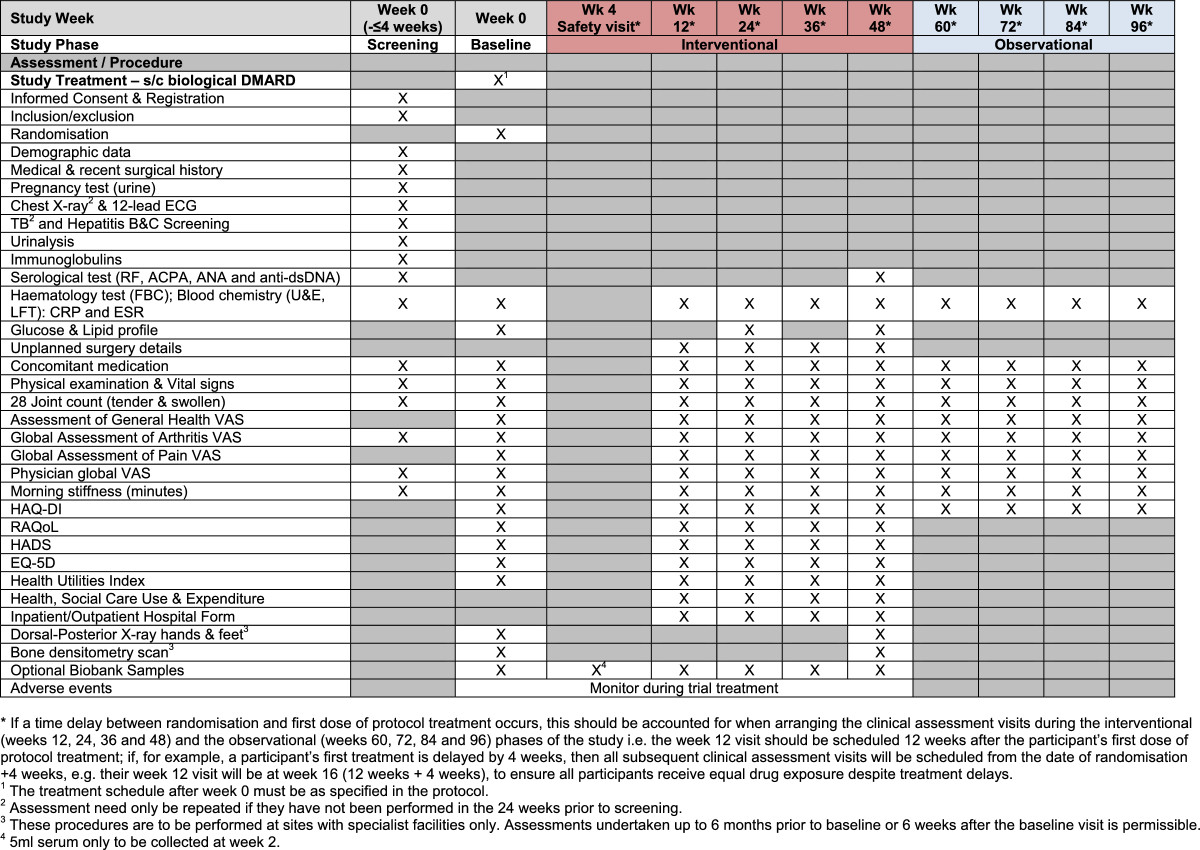
**Schedule of events for subcutaneous biologic DMARDs (etanercept, adalimumab, certolizumab pegol, abatacept, golimumab).**

All protocol-required assessments will be recorded on paper case report forms at each site.The trial visits are structured as detailed below (see also Figures 2,3 &4):

- Screening visit: All patients will undergo screening within 4 weeks prior to the baseline assessment.
- Baseline visit: Baseline assessments are to be performed to confirm that the participant is still eligible for the study and to undertake randomisation to study treatment.
- Clinical assessment visits: Randomised participants attend these visits as part of the interventional (weeks 12, 24, 36 and 48) and the observational (weeks 60, 72, 84 and 96) phases of the study.
- Infusion visits: Participants allocated to Rituximab or Infliximab will undergo additional standard assessments for safety purposes on the infusion dates.Biological samples from participants consenting to the SWITCH Trial BioBank sub-study will be collected prior to commencement of trial treatment and at weeks 2/4, 12, 24, 48 and at the time of early discontinuation if it occurs outside of these time-points (see Figures 2,3 &4). The samples will be sent to a central Switch Trial Biobank. These samples will be used for a range of studies of direct relevance to the treatment of RA.

### Outcomes

The primary outcome is the change in Disease Activity Score 28 (DAS28)[68] at 6 months (24 weeks). Secondary outcomes at weeks 12, 24, 36 and 48 are: the DAS28 score and the proportion of participants who achieve a reduction in DAS28 score of greater than 1.2 from baseline, Low Disease Activity Score (LDAS) rate[69] and remission rate[70], EULAR (European League Against Rheumatism) and ACR (American College of Rheumatology) response scores[69, 71], changes in scores and proportion of participants in each category of the Clinical Disease Activity Index (CDAI)[72] and Simplified Disease Activity Index (SDAI)[73], the proportion of participants that achieve ACR/EULAR Boolean remission rate[70] at each time-point. The outcomes relating to quality of life at weeks 12, 24, 36 and 48 are: the RA Quality of Life (RAQoL)[74], the Hospital Anxiety and Depression Scale (HADS)[75], and the Health Assessment Questionnaire Disability Index (HAQ-DI)[76]; the HAQ-DI will also be evaluated at weeks 60, 72, 84 and 96. The outcomes required for the cost-effectiveness analysis and collected at weeks 12, 24, 36 and 48 are: EQ-5D[77], Health Utilities Index (HUI)[78], Health and Social Care Use & Expenditure due to Rheumatoid Arthritis[79]. EQ-5D and Health Utilities Index will also be evaluated at weeks 60, 72, 84 and 96. Further outcomes correspond to safety (adverse events and reactions) and toxicity (requiring cessation of treatment) reported throughout the duration of the trial treatment (up to week 48). In addition, outcomes related to radiographic measures at week 48 will be: changes in Genant-Sharp scores[80] of hands and feet, and bone densitometry T-scores of neck of femur and lumbar spine.

### Sample size

A total of 477 participants will be recruited to this study. A total of 429 evaluable participants are required to have 80% power for demonstrating non- inferiority of either abatacept or alternative mechanism TNFi to rituximab at the 5% significance level. A total of 143 evaluable participants in each treatment group will ensure that the lower limit of the two-sided 95% confidence interval for the true difference in DAS28 (abatacept/alternative mechanism TNFi – rituximab) lies above -0.6 units, assuming no difference between treatment groups and a between-participant standard deviation of 1.8 units[44]. Allowing for a loss to follow-up of 10%, a total of 477 participants will be recruited.

### Statistical analysis

All analyses will be conducted on the Intention-To-Treat (ITT) population, where patients will be included according to the treatment to which they were allocated at randomisation. A Per-Protocol (PP) population will also be defined for the non-inferiority analyses, which will exclude participants who violate the protocol or fail to comply with the required treatment regime. Non-inferiority will need to be demonstrated in both ITT and PP populations in order to infer non-inferiority. All formal analyses will be carried out at a 2-sided 5% level of significance.

An interim analysis will be conducted after 239 participants have completed 24 weeks of follow-up to allow for early stopping of a treatment arm; specifically if either abatacept or alternative mechanism TNFi is shown to be inferior to rituximab, which will be based on the confidence interval excluding the value zero.

### Primary outcome analyses

Multiple-variable linear regression will be used to compare the alternative mechanism TNFi and abatacept to rituximab with the dependent variable, the change in DAS28 at 6 months (24 weeks), and the minimisation factors (centre, disease duration (<5 years, ≥5 years), rheumatoid factor status, primary/secondary non-response) and the baseline value of DAS28 included as independent variables. The mean treatment differences, 95% CIs and p-values from this analysis will provide the main comparisons for each treatment group with rituximab.

### Secondary outcome analyses

Alternative mechanism TNFi and abatacept will be compared to rituximab at 12, 24, 36 and 48 weeks using the following methods:

- DAS28: Multi-level repeated measures analysis, including minimisation factors and baseline DAS28 in addition to treatment.
- Markers of achieving DAS28 reduction of greater than 1.2 without toxicity, DAS28 LDAS and remission rates, ACR/EULAR Boolean remission and ACR response rates: Binary logistic regression analysis including the minimisation factors and baseline DAS28 in addition to treatment.
- EULAR response scores, SDAI and CDAI scores: Ordinal logistic regression analysis including the minimisation factors and baseline DAS28 in addition to treatment.
- RAQoL, HADS, HAQ-DI: Linear regression analysis will fitted to the change in QoL scores between baseline and 6 months including the minimisation factors and baseline DAS28 in addition to treatment.
- Safety and Toxicity: The proportion of participants experiencing toxicity will be summarised by treatment received. Adverse events (including serious adverse events (SAEs), serious suspected adverse reactions and suspected unexpected serious adverse events) will be summarised by treatment group and the relationship between events and study treatment or underlying RA will be assessed. Expected SAEs common to all treatments include injection site/infusion reactions, blood dyscrasias, serious infections, toxic epidermal necrolysis, Stevens-Johnson syndrome, pulmonary fibrosis, renal failure, neurological impairment, and new autoimmunity. In addition, intolerance to protocol treatment will be summarised by treatment received.

### Exploratory analyses

To determine if there is a differential response according to TNFi type (monoclonal antibody or fusion protein) initially failed, a linear regression model will be fitted to DAS28 at 24 weeks including baseline DAS28, type of TNFi initially received and minimisation factors as independent variables. To determine if there is a differential treatment effect according to primary and secondary failure to initial TNFi received, a linear regression model will be fitted to DAS28 at 24 weeks on baseline DAS28, treatment, primary/secondary failure, remaining minimisation factors, and an interaction term between treatment and type of failure. Finally a linear regression model will be fitted to DAS28 at 24 weeks including baseline DAS28, treatment, rheumatoid factor status, remaining minimisation factors, and an interaction term between treatment and rheumatoid factor status in order to assess if there is a differential treatment response between seropositive and seronegative patients.

### Economic evaluation

The economic evaluation aims to assess overall cost-effectiveness from the perspective of the health system (NHS) and patients. It will consist of a within-trial cost effectiveness analysis and a decision analytic cost effectiveness model. The within-trial analysis will evaluate the costs and outcomes of the patients recruited to SWITCH for the follow-up of the trial. As with the primary analysis, the economic evaluation will be an intention to treat analysis. The outcome used in the primary analysis will be the Quality Adjusted Life Year. Using the NICE cost effectiveness threshold of 20,000 per QALY[81], we will convert costs and outcomes for each patient on to the Net Benefit scale and use linear regression analysis to estimate the expected Net Benefit of the trial interventions compared to current practice[82]. Analysis of uncertainty will be undertaken using the non-parametric bootstrap, to characterise the uncertainty in the estimates of Net Benefit.

A second analysis will synthesise the data from the SWITCH trial with existing evidence to estimate the lifetime expected net benefit of the trial interventions compared to current standard care. The perspective for this analysis will be the same as for the within trial analysis. In order to capture the switching nature of the treatment pathways, we will construct patient level simulation model, rather than the Markov cohort model, which is frequently used for decision analytic cost effectiveness analyses.

The primary modelled analyses will adopt the perspective of the NHS and Public Social Services. Secondary analyses will adopt a broader perspective incorporating carer quality of life and cost impacts, and productivity costs.

Resource utilisation will be captured at each follow-up visit. Personal expenditures related to the management of RA, and time spent away from work by the patient and carers will be collected using the Cost Diary, a validated questionnaire[79]. Unit costs will be taken from routine national databases such as the British National Formulary, the NHS Reference Costs and the Personal Social Services Research Unit (PSSRU) costs of health and social care[83]. Health-related quality of life will be captured using the EQ-5D supplemented by the Health Utilities Index. These data will be collected at baseline and at each clinical follow-up. Parameter uncertainty will be addressed through probabilistic sensitivity analysis. For the within trial analysis this will be done using the non-parametric bootstrap; for the decision analytic cost effectiveness model this will be done using Monte Carlo simulation.

Outputs from the analyses will be presented as Expected Incremental Cost Effectiveness Ratios; Cost Effectiveness Acceptability Frontiers, Expected Net Benefit[84] and Net Benefit Probability Maps[85]. In addition to the primary analyses, secondary analyses adopting different perspectives, different utility measures and different approaches to dealing with missing data will be presented. The final set of analyses will present estimates of the global and partial value of perfect information, to inform future research.

## Discussion

RA has a substantial individual and societal burden: symptoms impact heavily on patients' ability to perform daily activities at home and ability to undertake work commitments with subsequent cost to the NHS and state. It is therefore important to treat this potentially disabling and expensively managed condition effectively and with the minimum of time delay.

There have been dramatic advances in the development of effective drugs to treat RA and the use of TNFi has transformed the lives of people suffering from RA. While these drugs can be highly effective, universal response has not been observed; indeed this is a common feature of all the available and licensed bDMARDs (alternative TNFi, rituximab, abatacept and most recently, tocilizumab) likely reflecting the complexity and heterogeneity of disease pathogenesis. Some observational studies and preliminary data from recent RCTs suggest both similar and better efficacy amongst the available classes of bDMARD but with no definitive investigation on the sequential biologic treatment strategy: making it difficult to draw any firm conclusion. Nevertheless, the National Institute for Health and Care Excellence (NICE) has approved only the use of rituximab following TNFi failure, thereby offering only one option to patients. SWITCH is a direct comparison trial that will facilitate the development of a rational treatment algorithm and should enable more judicious and cost-effective management. In addition, the exploratory analyses in this trial may provide information on more effective targeting of treatment regimens, as opposed to switching all patients to only one available (and possible unsuccessful) therapy (rituximab), leading to long-term cost-benefits and improved patient outcomes.

### Trial status

The first patient was enrolled into SWITCH on the 31st July 2012 and recruitment is due to end in December 2016. The study is being conducted in multiple NHS sites across the UK, with a planned total of up to 50 sites. We expect to report the main trial results in Autumn 2018. Ethical and governance approval for this trial has been obtained from the Leeds West Ethics Committee (ref 11/H1307/6) and the Leeds Teaching Hospitals NHS Trust respectively. The trial progress is monitored by an independent Data Monitoring and Ethics Committee (DMEC) and Trial Steering Committee (TSC).

Since opening, the trial has undergone a major trial re-design. Our original target sample size was 870 patients, to have 80% power for determining whether abatacept or alternative TNFi were non-inferior to rituximab at 24 weeks post randomisation in terms of achieving a DAS28 reduction of greater than 1.2 points without toxicity. The corresponding non-inferiority margin was set at 12% and assumed a response rate of 65% in the rituximab arm. The original trial design was also powered for a definitive sub-group analysis to determine if there is a differential treatment response between seropositive and seronegative patients Following challenges in recruiting patients and securing site participation, as well as re-discussion of meaningful endpoints, a decision was made to re-design the trial by modifying the primary outcome measure from a binary to a continuous outcome (which was also deemed clinically relevant). This allowed a reduction in sample size to 477 patients whilst still ensuring a trial of clinical relevance. The previous planned definitive sub-group analysis is now an exploratory analysis. The trial re-design was unanimously supported by the DMEC and the TSC, approved by the funder and has received favourable ethical opinion.

## Authors’ original submitted files for images

Below are the links to the authors’ original submitted files for images. Authors’ original file for figure 1 Authors’ original file for figure 2 Authors’ original file for figure 3 Authors’ original file for figure 4

## Abbreviations

ACPA: Anti-citrullinated peptide antibody
ACR: American college of rheumatology
AIA CSG: Adult inflammatory arthritis clinical studies group
AUC: Area under the curve
bDMARD: biologic disease modifying anti-rheumatic drug
BSR: British society of rheumatology
CDAI: Clinical disease activity index
CI: Confidence interval
CIGMR: Centre for integrated genomic medical research
CCP: Cyclic citrullinated peptide
CTRU: Clinical trials research unit
DAS: Disease activity score
DMARD: Disease modifying anti-rheumatic drug
DMEC: Data monitoring & ethics committee
EQ-5D EuroQol: 5 Dimensions questionnaire
EULAR: European league against rheumatism
HADS: Hospital anxiety and depression scale
HAQ-DI: Health assessment questionnaire - disability index
ITT: Intention-to-treat
LDA: Low disease activity
Mg: Milligrams
NHS: National health service
NICE: National institute for health and care excellence
PIC: Participant identification centre
PSSRU: Personal social services research unit
QALY: Quality adjusted life year
RA: Rheumatoid arthritis
RAQoL: Rheumatoid arthritis quality of life
RF: Rheumatoid factor
RCT: Randomised controlled trial
SDAI: Simplified disease activity index
SMR: Standardised mortality rate
TNF: Tumour necrosis factor (blocking agents also referred to as TNF antagonists)
TNFi: TNF inhibitor
TSC: Trial steering committee.
