# Supplementary figures and images for: The 'Switch’ study protocol: a randomised-controlled trial of switching to an alternative tumour-necrosis factor (TNF)-inhibitor drug or abatacept or rituximab in patients with rheumatoid arthritis who have failed an initial TNF-inhibitor drug
Source: BMC Musculoskelet Disord. 2014 Dec 23;15:452. doi: 10.1186/1471-2474-15-452 (PMC4391115; doi:10.1186/1471-2474-15-452)

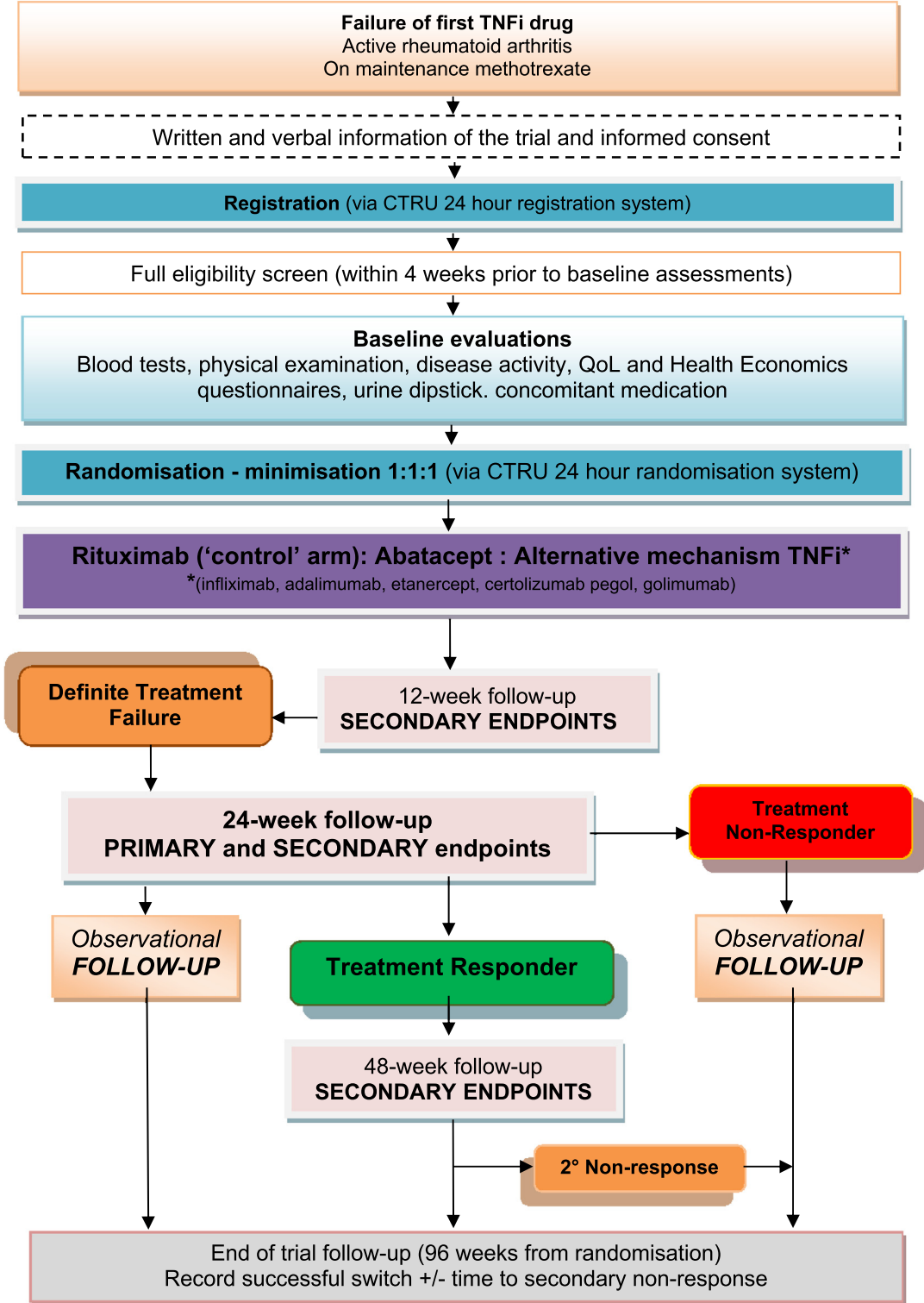

Supplement: Supplementary file 1 — Authors’ original file for figure 1 [file 12891_2014_2445_MOESM1_ESM.pdf]
