# Supplementary material for: The 'Switch’ study protocol: a randomised-controlled trial of switching to an alternative tumour-necrosis factor (TNF)-inhibitor drug or abatacept or rituximab in patients with rheumatoid arthritis who have failed an initial TNF-inhibitor drug
Source: BMC Musculoskelet Disord. 2014 Dec 23;15:452. doi: 10.1186/1471-2474-15-452 (PMC4391115; doi:10.1186/1471-2474-15-452)
Supplement: Supplementary file 4 — Authors’ original file for figure 4 [file 12891_2014_2445_MOESM4_ESM.pdf]

| Study Week | Week 0 (-≤4 weeks) | Week 0 | Wk 4 Safety visit* | Wk 12* | Wk 24* | Wk 36* | Wk 48* | Wk 60* | Wk 72* | Wk 84* | Wk 96* |
|---|---|---|---|---|---|---|---|---|---|---|---|
| **Study Phase** | **Screening** | **Baseline** | **Interventional** | | | | | **Observational** | | | |
| **Assessment / Procedure** | | | | | | | | | | | |
| **Study Treatment – s/c biological DMARD** | | X[1] | | | | | | | | | |
| Informed Consent & Registration | X | | | | | | | | | | |
| Inclusion/exclusion | X | | | | | | | | | | |
| Randomisation | | X | | | | | | | | | |
| Demographic data | X | | | | | | | | | | |
| Medical & recent surgical history | X | | | | | | | | | | |
| Pregnancy test (urine) | X | | | | | | | | | | |
| Chest X-ray[2] & 12-lead ECG | X | | | | | | | | | | |
| TB[2] and Hepatitis B&C Screening | X | | | | | | | | | | |
| Urinalysis | X | | | | | | | | | | |
| Immunoglobulins | X | | | | | | | | | | |
| Serological test (RF, ACPA, ANA and anti-dsDNA) | X | | | | | | X | | | | |
| Haematology test (FBC); Blood chemistry (U&E, LFT): CRP and ESR | X | X | | X | X | X | X | X | X | X | X |
| Glucose & Lipid profile | | X | | | X | | X | | | | |
| Unplanned surgery details | | | | X | X | X | X | | | | |
| Concomitant medication | X | X | | X | X | X | X | X | X | X | X |
| Physical examination & Vital signs | X | X | | X | X | X | X | X | X | X | X |
| 28 Joint count (tender & swollen) | X | X | | X | X | X | X | X | X | X | X |
| Assessment of General Health VAS | | X | | X | X | X | X | X | X | X | X |
| Global Assessment of Arthritis VAS | X | X | | X | X | X | X | X | X | X | X |
| Global Assessment of Pain VAS | | X | | X | X | X | X | X | X | X | X |
| Physician global VAS | X | X | | X | X | X | X | X | X | X | X |
| Morning stiffness (minutes) | X | X | | X | X | X | X | X | X | X | X |
| HAQ-DI | | X | | X | X | X | X | X | X | X | X |
| RAQoL | | X | | X | X | X | X | | | | |
| HADS | | X | | X | X | X | X | | | | |
| EQ-5D | | X | | X | X | X | X | | | | |
| Health Utilities Index | | X | | X | X | X | X | | | | |
| Health, Social Care Use & Expenditure | | | | X | X | X | X | | | | |
| Inpatient/Outpatient Hospital Form | | | | X | X | X | X | | | | |
| Dorsal-Posterior X-ray hands & feet[3] | | X | | | | | X | | | | |
| Bone densitometry scan[3] | | X | | | | | X | | | | |
| Optional Biobank Samples | | X | X[4] | X | X | X | X | | | | |
| Adverse events | | Monitor during trial treatment | | | | | | | | | |

* If a time delay between randomisation and first dose of protocol treatment occurs, this should be accounted for when arranging the clinical assessment visits during the interventional (weeks 12, 24, 36 and 48) and the observational (weeks 60, 72, 84 and 96) phases of the study i.e. the week 12 visit should be scheduled 12 weeks after the participant's first dose of protocol treatment; if, for example, a participant's first treatment is delayed by 4 weeks, then all subsequent clinical assessment visits will be scheduled from the date of randomisation +4 weeks, e.g. their week 12 visit will be at week 16 (12 weeks + 4 weeks), to ensure all participants receive equal drug exposure despite treatment delays.

[1] The treatment schedule after week 0 must be as specified in the protocol.

[2] Assessment need only be repeated if they have not been performed in the 24 weeks prior to screening.

[3] These procedures are to be performed at sites with specialist facilities only. Assessments undertaken up to 6 months prior to baseline or 6 weeks after the baseline visit is permissible.

[4] 5ml serum only to be collected at week 2.
